# Mutations of *nhr-49* affect *C. elegans* susceptibility to *Yersinia* biofilms

**DOI:** 10.17912/micropub.biology.001522

**Published:** 2025-02-04

**Authors:** Jonathan Hodgkin, Dave Stroud, Delia O'Rourke

**Affiliations:** 1 Biochemistry, University of Oxford, Oxford, England, United Kingdom; 2 Centre for Human Genetics, University of Oxford, Oxford, England, United Kingdom

## Abstract

The *C. elegans* transcription factor NHR-49 has been extensively studied for its functions in regulating metabolic processes, stress responses, innate immunity and aging. Molecular identification of a gene previously known as *bah-3* , which affects susceptibility of worms to deleterious surface attachment of bacterial biofilms from *Yersinia spp.,* revealed that *bah-3 ( dc9 )* is an ochre nonsense allele of *nhr-49* . Other severe mutations of *nhr-49* also had a Bah phenotype, but deletions affecting 5' isoforms of the gene did not affect biofilm attachment, nor did 3' gain-of-function missense mutations. Other *bah* genes ( *bah-1 , bah-2 , bah-4* ) encode GT92 glycosylation factors, predicted to affect surface coat. NHR-49 may act as a positive transcription factor for one or more of these surface glycosylation genes, in contrast to its other roles in regulating metabolic processes.

**Figure 1. The Bah phenotype affected by bah-2 and nhr-49:**
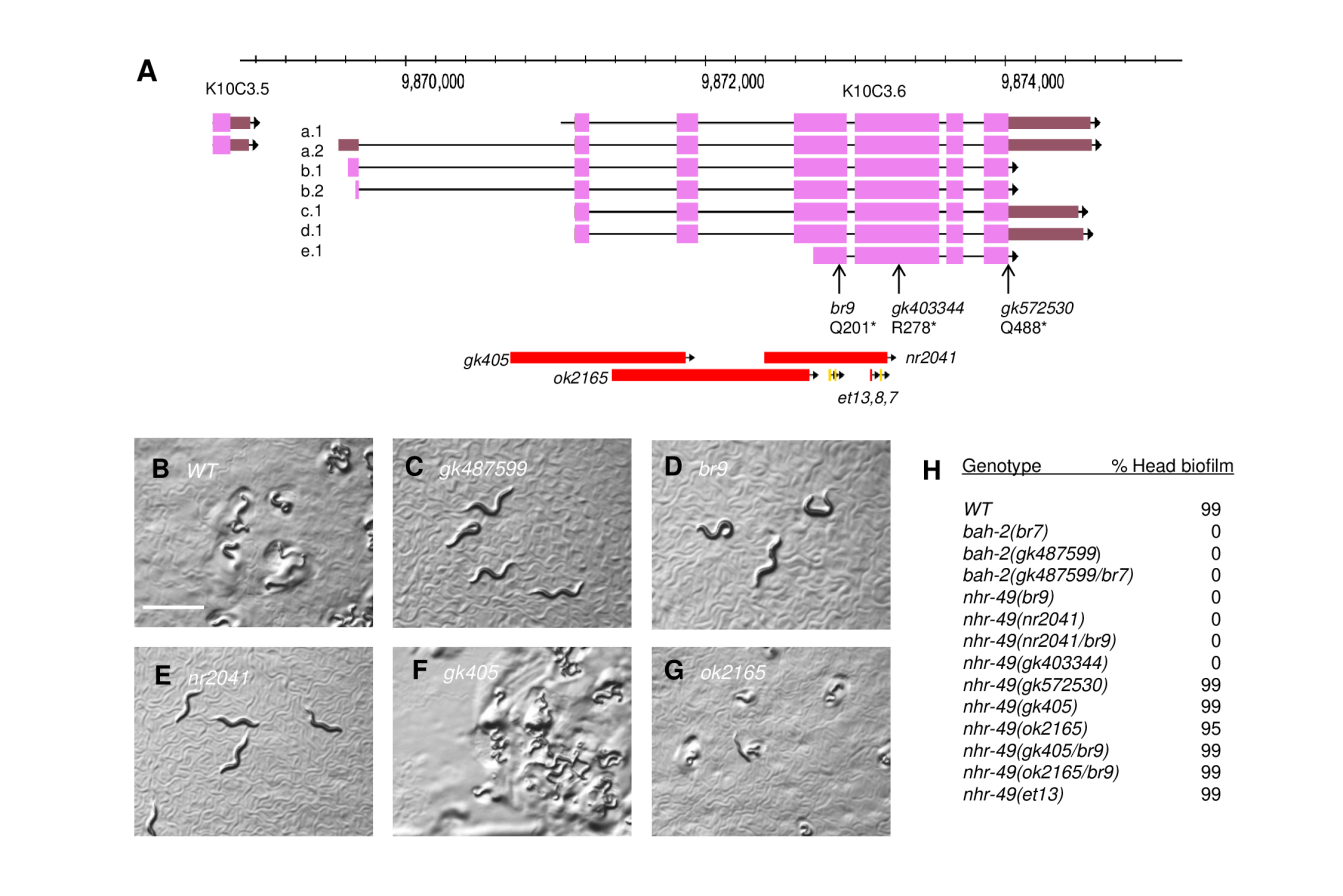
A) Genome organization and mutations of gene *nhr-49* ( K10C3.6 ); image modified from WormBase (Sternberg et al., 2024). Deletion extents are 1275bp ( *gk405* ), 1437bp ( *ok1265* ), 893bp ( *nr2041* ). Mutation locations are given for the longest NHR-49 isoform (isoform c: 501aa). Missense mutations (Svensk et al., 2013) are *et13* (V387E), *e8* (S408F), *et7* (P455L). B-G) Photographs of larval worms, 14 – 24 hours after hatching at 25˚ on YPIII lawns. Non-Bah worms (Figure B, F, G) arrested at L1 with head biofilm and poor movement; Bah worms (Figure C, D, E) were unaffected. Uniform magnification for all photographs; scale bar in B: 0.5 mm. H) Percentage biofilm formation on larval heads after growth on YPIII. All Bah tests (rows 2-8) were significantly different (p < 0.0001) from wild type (row 1). At least 100 larvae were scored for each test.

## Description

The *Caenorhabditis nhr* gene class comprises 283 members in the *Caenorhabditis elegans* genome, encoding transcription factors of the NHR (Nuclear Hormone Receptor) family. Of these, *nhr-49* ( K10C3.5 , **Figure 1A** ) has been implicated in regulating a remarkable variety of metabolic processes (reviewed by Doering & Taubert, 2023, Sala et al., 2024). Both loss-of-function and gain-of-function mutations have been studied. *C. elegans* NHR-49 is an ortholog of mammalian HNF4/PPARalpha, with diverse roles in regulating lipid metabolism, oxidative and heat stress responses, fasting, innate immunity and longevity, acting in many different tissues. However, effects on nematode surface properties have not been reported hitherto.

*C. elegans bah* mutants were defined by Darby et al., (2007), as being resistant to the formation of deleterious biofilms on the surface of young larvae exposed to strains of either *Yersinia pestis* (the causative agent of bubonic plague) or *Yersinia pseudotuberculosis* (mildly pathogenic to humans). Wildtype worms hatching on lawns of YPIII (a standard *Y. pseudotuberculosis* strain) accumulated head biofilms that impaired feeding and growth (phenotype Bah, for Biofilm Absent on Head: **Figure 1B** ). In contrast *bah* mutants were unencumbered, with growth rates on pure YPIII lawns similar to growth on standard *E. coli* OP50 food ( **Figure 1C, 1D** ). Initial studies defined three *bah* genes; *bah-1* was subsequently identified as encoding a predicted GT92 glycosyltransferase (Hansen et al., 2012) , expressed in seam cells (Drace et al., 2009) .

In this work, *bah-2* was identified as another member of the large class of *C. elegans* GT92 glycosyltransferase genes (62 members). Whole-genome sequencing (Sarin et al., 2008) of a *bah-2 ( br7 )* strain indicated that this strain carried a missense mutation (Asp304Asn) in the GT92-encoding gene F18F11.4 at the predicted genomic location of *bah-2* . A nonsense mutation of F18F11.4 , *gk487599* (Trp231*), has been generated by the Million Mutation Project (Thompson et al., 2013) . This mutation failed to complement *bah-2 ( br7 ),* and the associated Bah phenotype was found to be fully rescued by a *bah-2 (+)* transgene. Repeated back-crossing against wild-type yielded a homozygous *bah-2 ( gk487599 )* strain, indistinguishable from *bah-2 ( br7 )* ( **Figure 1C** ).

The same strategy was used to identify the molecular nature of *bah-3 ( br9 )* ( **Figure 1D** ). Surprisingly, *br9* strains were found to carry an ochre nonsense mutation (Gln201*) in *nhr-49* , which has a similar genetic location to that previously established for *bah-3* (Darby et al., 2007) . The deletion mutation *nr2041* , which has been used as a standard null mutant for *nhr-49* (Van Gilst et al., 2005) , was examined and found to exhibit a Bah phenotype, as did *nr2041 / br9* trans-heterozygotes ( **Figure 1E, 1H** ). Two nonsense mutations of *nhr-49* generated by the Million Mutation Project were also tested after out-crossing: *gk403344* (Arg131*) homozygotes were Bah, but *gk572530* (Gln488*) homozygotes were not ( **Figure 1A, 1H** ). However, the latter lesion would result in the loss of only 14 residues at the C-terminus of NHR-49 , and therefore may have little effect on NHR-49 function.

Genomic and transcriptomic studies (summarized on WormBase, Sternberg et al., 2024) indicate that *nhr-49* has a complex structure, with at least seven transcripts encoding five protein isoforms ( **Figure 1A** ), which differ mainly in the inclusion of 5' exons. Two deletion mutations ( *gk405 , ok2165 :* Figure 1A ) are predicted to affect most of these isoforms (a,b,c,d) but they were found to have little or no effect on the Bah phenotype ( **Figure 1F, 1G, 1H** ). The deletion allele *ok2165* has a break point 210 bp upstream of the first exon of isoform e, so expression of this isoform may be unaffected. Therefore, it is probable that isoform e (a 354 aa protein with homology to human Hepatocyte nuclear factor 4-gamma), is the only Bah-relevant product of *nhr-49* . All Bah mutations of *nhr-49* affect isoform e. In addition, three gain-of-function missense mutations ( et7 , et8 , et13 ) affecting all isoforms have been examined. These were isolated as suppressors of the cold-sensitivity of *paqr-2* mutants but differ in their effects on longevity (Svensk et al., 2013, Lee et al., 2016) . The corresponding mutants were tested on YPIII lawns, but were found to have a wild type response ( **Figure 1H** ).

Four other genes ( *nhr-13 , nhr-66 , nhr-80 , mdt-15* ) have been identified as encoding transcriptional co-factors for some but not all NHR-49 functions (Pathare et al., 2012, Taubert et al., 2006, Goh et al., 2014) . Single and double mutants for these factors were tested on YPIII lawns and all were found to accumulate biofilm, indicating that none of these factors is required by NHR-49 for this response (mutant strains listed in Methods).

A simple explanation for the effect of *nhr-49 ( br9 )* on YPIII biofilm formation is that NHR-49 (isoform e) is necessary for the expression of one or more of the *bah* genes, which probably affect the synthesis of surface glycans that recruit *Yersinia* biofilm. Both *bah-1* and *bah-2* encode GT92 glycosyltransferases. Several other uncharacterized GT92 genes have been tested for involvement in biofilm formation, using available deletion or nonsense mutations. Of 18 tested genes, a deletion affecting F13G3.3 was found to result in a Bah phenotype, so F13G3.3 is tentatively identified as *bah-4* . However, a nonsense mutant of T09E11.8 , which encodes a close paralog of *bah-2* , did not exhibit a Bah phenotype, nor did probable null mutants of the other 16 genes (strains listed in Methods).

At least eight other surface glycosylation genes also affect Yersinia biofilm formation ( *srf-2 , srf-3 , bus-2 , bus-4 , bus-5 , bus-12 , bus-17 , bus-22* ) as previously reported (Darby et al., 2007, O'Rourke et al., 2023) but these genes are unlikely to be targets for *nhr-49* regulation because *nhr-49 ( br9 )* mutants do not exhibit altered responses to *Microbacterium* and *Leucobacter* bacterial pathogens, unlike the mutants of these *bus* and *srf* genes (Hodgkin et al. 2013, O'Rourke et al., 2023). The biofilm effect of *nhr-49* therefore seems to be specific to GT92 *bah* genes. A survey of differentially regulated genes in *nhr-49* mutants did not identify any of *bah-1 , bah-2* or *bah-4* as a major target for NHR-49 regulation (Pathare et al., 2012) . However, effects on transcription of these *bah* genes might be less easily detected than effects on the many metabolic targets of NHR-49 .

Genes affecting surface properties are primarily expressed in ectodermal tissues (seam cells and hypodermis) (Gravato-Nobre et al., 2011, O'Rourke et al., 2023) which are among the many tissues where *nhr-49* is known to be expressed (Taubert et al. 2006) , so an additional role for this gene in regulating surface properties is possible though unexpected. The Bah phenotype of *nhr-49* null mutants is dramatic and can be detected within a few hours of worm growth on *Yersinia* lawns; it may therefore provide a convenient assay for rapidly assessing NHR-49 function *in vivo* .

## Methods

1. Culture

Standard methods for *C. elegans* culture were used (Brenner 1984) . Mutant strains were grown up and tested at 25˚C.

2. Biofilm assessment

For head biofilm assessment, *Yersinia pseudotuberculosis* strain YPIII (Gemski et al., 1980) was grown to stationary phase in LB broth. 50 ml spots were placed on NGM plates and incubated overnight at 25˚C. At least 100 eggs from each tested strain were placed on the resulting YPIII lawns and incubated for 24 hours at 25˚. Presence of head biofilm on at least 100 hatchling larvae (L1 – L2) was then counted and expressed as a percentage ( Figure 1H ). Tests were carried out at least three times with independent preparation of YPIII lawns, always with a wild type control and always with similar results.

3. Transgenic rescue of *bah-2*

An operon construct consisting of 4.4 kb including F18F11.4 and 865 bp of upstream sequence plus 3' TagRFP-T was combined with *unc-119 (+)* and injected into *unc-119 ( ed3 )* worms to generate *eEx835* , which was then crossed into *bah-2 ; unc-119 ( ed3 )* worms. Both *bah-2 ( br7 )* and *bah-2 ( gk487599 )* strains were fully rescued. RFP fluorescence was observed in seam cells.

## Reagents

Strain Genotype Origin

N2 wildtype Lab CB

DC7 *bah-2 ( br7 )* Lab DC

DC9 *nhr-49 ( dc9 )* Lab DC

CB7201 *bah-2 ( gk487599 )* CGC/ VC40154

CB7401 *bah-2 ( gk487599 ); unc-119 ( ed3 ); eEx835* Lab CB

CB7589 *nhr-40 ( gk403344 )* CGC/ VC20789

CB7590 *nhr-40 ( gk572530 )* CGC/ VC40321

CB7396 *nhr-49 ( et7 )* CGC/ QC120

CB7397 *nhr-49 ( et8 )* CGC/ QC121

CB7398 *nhr-49 ( et13 )* CGC/ QC126

RB1716 *nhr-49 ( ok2165 )* CGC

STE68 *nhr-49 ( nr2041* ) CGC

STE69 *nhr-66 ( ok940* ) CGC

STE72 *nhr-80 ( tm1011 ); nhr-66 ( ok940 )* CGC

STE70 *nhr-80 ( tm1011 )* CGC

STE71 *nhr-13 ( gk796 )* CGC

STE73 *nhr-13 ( gk796 ); nhr-80 ( tm1011 )* CGC

VC870 *nhr-49 ( gk405 )* CGC

XA7702 *mdt-15 ( tm2182 )* CGC

GT92 knockout or nonsense strains tested for Bah phenotype; each GT92 allele is given in parenthesis:

VC20280 : C13A2.6 *( gk239003 );* VC20176 C14C6.6 *( gk224770 );*

VC40439 C14C6.8 *( gk638318 );* VC20204 : C33H5.2 ( *gk206142 );*

RB664 : F13G3.3 ( *ok416 );* VC20221 : F39G3.2 *( gk233591 );*

VC40511 : F54D10.8 *( gk671679 );* VC20331 : F55C10.4 *( gk247338 );*

VC20661 : R05A10.6 *( gk217775 );* VC20320 : R07B7.12 ( *gk248807 );*

VC40841 : T09E11.8 *( gk841550 );* VC40942 : T15D6.10 *( gk958187 );*

VC30241 : T15D6.12 *( gk445311 );* VC40301 : T22D1.1 *( gk562447 );*

VC20674 : Y18H1A.14 *( gk101420 );* VC40950 : Y105C5B.25 *( gk896211 );*

VC20251 : ZK381.18 *( gk204592 );* VC30194 : ZK488.6 *( gk434742 )*
